# Premature mortality and disparities in kidney healthcare for people with chronic kidney disease and severe mental health difficulties

**DOI:** 10.1007/s40620-024-02103-6

**Published:** 2024-11-02

**Authors:** Clodagh Cogley, Mimi Smith-Jones, Elizabeth R. Ralston, Jessica Bramham, Joseph Chilcot, Paul D’Alton, Claire Carswell, Chun Chiang Sin Fai Lam, Ashutosh Ratnam, Mohammad Al-Agil, Hugh Cairns, Kufreabasi Imo Etuk, Kate Bramham

**Affiliations:** 1 School of Psychology, University College Dublin, Newman Building, Dublin 4, Ireland; 2 Department of Psychology, Institute of Psychiatry, Psychology and Neuroscience, King’s College London, London, UK; 3 Department of Women and Children’s Health, King’s College London, London, UK; 4 UK Health Security Agency, London, England, UK; 5 Department of Health Sciences, University of York, Heslington, York UK; 6 King’s College Hospital NHS Trust, London, UK

**Keywords:** Kidney disease, Mental health, Severe mental illness, Healthcare access

## Abstract

**Background:**

People with severe mental health difficulties, including schizophrenia, bipolar disorder and psychosis, have higher risk of chronic kidney disease (CKD). Little was known regarding clinical outcomes and utilisation of kidney care for people with CKD and severe mental health difficulties.

**Methods:**

We conducted a retrospective cohort analysis of individuals with CKD attending a tertiary renal unit in London, between 2006 and 2019. Individuals with severe mental health difficulty diagnoses were identified, and differences between those with and without severe mental health difficulties were analysed.

**Results:**

Of the 5105 individuals with CKD, 112 (2.2%) had a recorded severe mental health difficulty diagnosis. The mean lifespan of those with severe mental health difficulties was 13.1 years shorter than those without severe mental health difficulties, *t*(1269) = 5.752, *p* < 0.001. People with severe mental health difficulties had more advanced CKD at their first nephrology appointment. There were no statistically significant differences between groups in the rates of kidney failure, age at onset of kidney failure, or time elapsed between first appointment and death/kidney failure. The number of inpatient admissions was similar between groups, but those with severe mental health difficulties had higher rates of emergency and ICU admissions. Among individuals on renal replacement therapy (RRT), those with severe mental health difficulties were less likely to receive a kidney transplant and peritoneal dialysis. For patients receiving haemodialysis, those with severe mental health difficulties had a higher proportion of shortened sessions, greater mean weight loss during sessions, and a higher proportion of serum potassium and phosphate levels outside normal ranges.

**Conclusions:**

Findings illustrate a number of disparities in kidney healthcare between people with and without severe mental health difficulties, underscoring the need for interventions which prevent premature mortality and improve kidney care for this population.

**Graphic Abstract:**

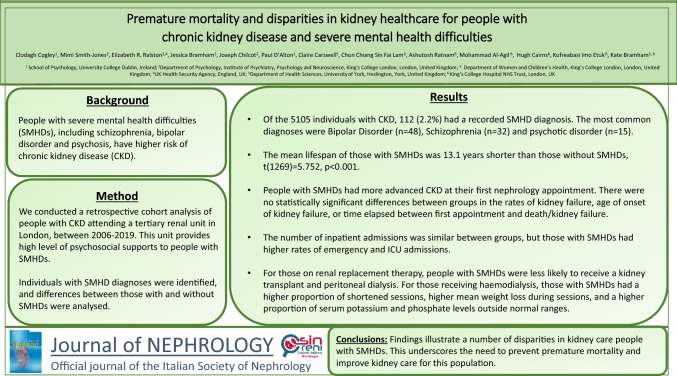

**Supplementary Information:**

The online version contains supplementary material available at 10.1007/s40620-024-02103-6.

## Introduction

People with severe mental health difficulties, including schizophrenia, bipolar disorder, and psychosis, have higher rates of chronic kidney disease (CKD) [1, 2]. This may be attributed to the use of psychiatric medications such as lithium, as well as higher rates of cardiovascular disease, diabetes, and smoking [3, 4]. In the United States (US), rates of psychiatric hospitalisations in people with kidney failure are 1.5–3 times higher than those with other chronic illnesses, and 27% of adults with kidney failure have had hospitalisations with a psychiatric diagnosis [6]. However, as these studies are based solely on data from inpatients with kidney failure, the true prevalence of severe mental health difficulties in people with CKD is unknown.

For people with CKD, research indicates those with concurrent severe mental health difficulties have a higher risk of mortality [6, 7]. In a nationwide cohort study in Taiwan, people with CKD and schizophrenia had a 23% higher risk of death [7], while among patients on haemodialysis, those with schizophrenia had an 84% higher risk of death over 5 years [7]. In the US, people with kidney failure who have been hospitalised with a psychiatric diagnosis had an increased risk of 1-year mortality compared to those hospitalised without a psychiatric diagnosis [6]. People with CKD and concurrent severe mental health difficulties also have higher hospitalisation rates, particularly through the emergency department, compared to those without severe mental health difficulties [8].

There is evidence that people with severe mental health difficulties receive suboptimal kidney care [4, 7, 9–11]. For example, people with severe mental health difficulties are less likely to receive a kidney transplant [1], despite evidence that individuals with severe mental health difficulties have similar post-transplant outcomes [12, 13]. People with severe mental health difficulties and kidney failure also have fewer appointments with nephrologists and are less likely to be scheduled for an evaluation for kidney transplant [7, 14]. Depressive symptoms are associated with missed and abbreviated haemodialysis sessions [15] and lower rates of dietary and fluid adherence [16]. However, little is known regarding rates of adherence and attendance for people with CKD and severe mental health difficulties.

To better understand the disparities in kidney care for people with severe mental health difficulties we conducted a retrospective cohort analysis of individuals attending a tertiary renal unit. Our research questions were:

1. What are the rates of severe mental health difficulty diagnoses in people with CKD?
2. What are the rates of psychotropic drug prescriptions in people with CKD?
3. In people with CKD, what is the difference in lifespan between those with and without a severe mental health difficulty diagnosis?
4. In people with CKD, do those with severe mental health difficulties differ demographically from those without severe mental health difficulties?
5. Is there a statistically significant difference between individuals with and without severe mental health difficulties with regard to the time elapsed between the first nephrology clinic appointment and the occurrence of kidney failure or death?
6. Do people with severe mental health difficulties differ in their utilisation of kidney care, compared to those without severe mental health difficulties? This will be assessed using (a) clinical data at the time of their first nephrology appointment, (b) hospital inpatient admissions, (c) renal replacement therapy (RRT) modalities, (d) clinic appointment attendance, and (e) adherence to haemodialysis.

## Methods

### Design and setting

This was a retrospective cohort analysis of patients with CKD attending a tertiary renal unit in London providing adult nephrology, renal transplant, and dialysis clinic services to a diverse patient population. The unit is responsible for the care of approximately 1200 patients with kidney failure, of whom 500 have functioning kidney transplants, 600 are on haemodialysis (in-centre, satellite and home haemodialysis), and 100 are on peritoneal dialysis. An Advanced Kidney Care Clinic (for those with eGFR < 20 mls/min) provides care to approximately 450 patients not receiving RRT. The study design was informed by a panel of three individuals with CKD and concurrent severe mental health difficulties.

The London South East Research ethics committee granted approval, with Caldicott Guardian oversight (22/SC/0136). As the study was retrospective and using de-identified data, informed consent was waived. Individuals who opted out of having their healthcare data used for research purposes were removed.

### Cohort selection and identification

**Fig. 1:**
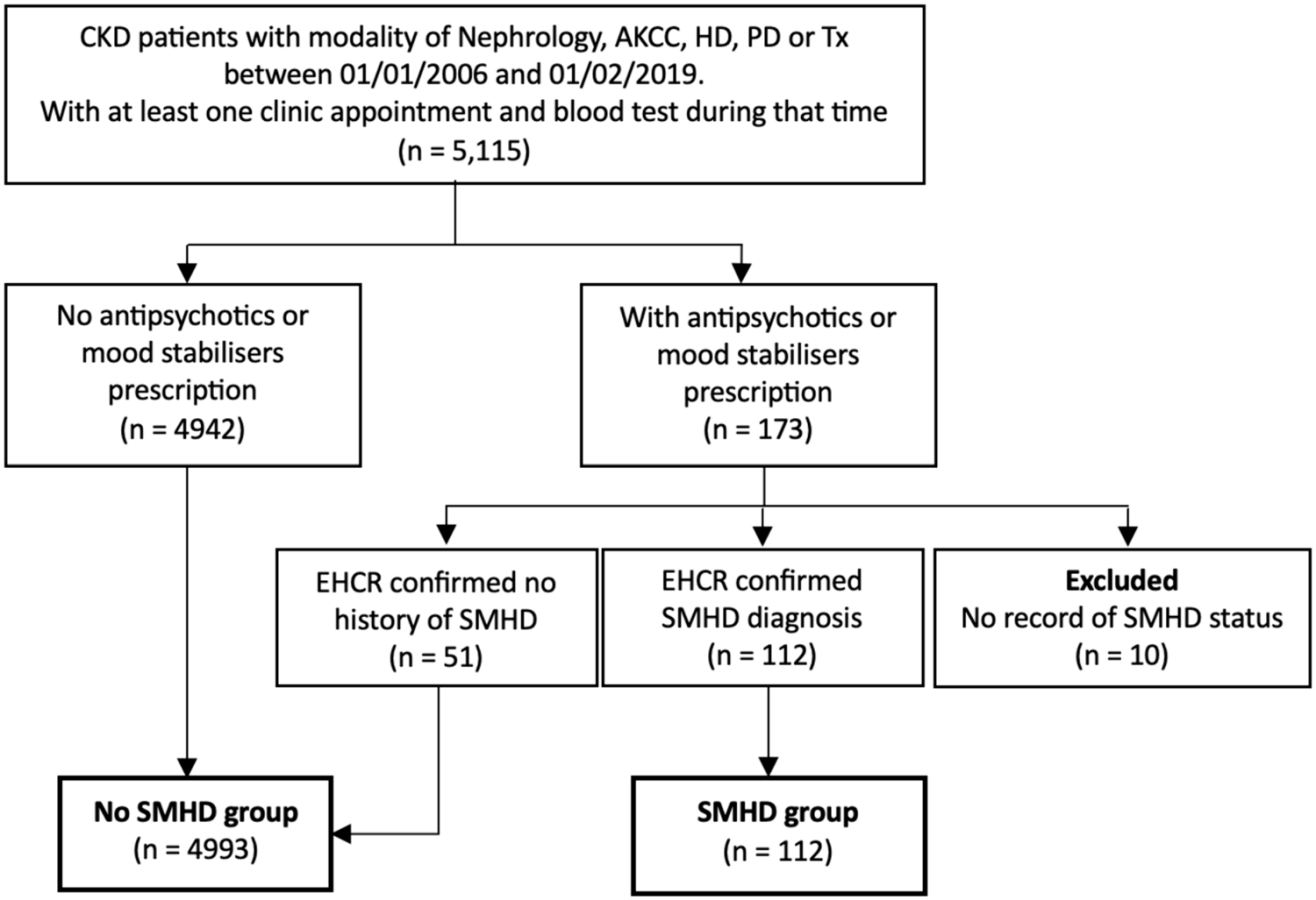
Cohort selection of individuals with CKD, with and without SMHD diagnoses. AKCC = advanced kidney care clinic; CKD = chronic kidney disease; HD = haemodialysis; PD = peritoneal dialysis; SMHD = severe mental health difficulty; Tx = Transplant

RenalWare^®^ Nephrology database was used for data extraction. See Fig. 1 for details of cohort selection. The timeframe was selected because Renalware transitioned to an open-source system in 2006, and healthcare utilisation patterns changed significantly during the COVID-19 pandemic in 2020.

Severe mental health difficulties were defined as encompassing schizophrenia, schizoaffective disorder, bipolar disorder, depression with psychotic features, and all other non-organic psychoses. Individuals who potentially had severe mental health difficulties were identified based on the prescription of antipsychotics and mood stabilisers. To confirm severe mental health difficulty diagnoses, we used the CogStack informatics platform to retrieve mentions of severe mental health difficulties, epilepsy and dementia from electronic healthcare records (EHCRs) (See supplementary materials for list of searched terms) [17]. Individuals without a severe mental health difficulty diagnosis recorded in their EHCRs were excluded from the severe mental health difficulty group (see Fig. 1).

### Extraction of demographic characteristics and outcome variables

Demographic and outcome variables were extracted from RenalWare and from EHCRs using Cogstack. For a full list of variables and how they were measured, see Supplementary Table II.

### Statistical analyses

A priori power analysis was calculated using G*Power3 (Version 3.1.9.4), for testing difference in lifespan between two groups using a one-tailed *t*-test, alpha of 0.05 and small effect size (Cohen’s *d* = 0.2). With an allocation ratio of 1:38, the sample size was sufficient to achieve a power of 0.80. Other statistical analyses were performed using IBM SPSS, version 28. Continuous variables with normal distribution are expressed as means and standard deviations, variables with skewed distribution are expressed as medians and interquartile ranges. See Supplementary Table II for details of variables used.

No systematic differences in outlier distribution were found between those with and without severe mental health difficulties. Clinically improbable values were removed (see supplementary Table I for valid ranges). For parametric tests, values exceeding three standard deviations from the mean were eliminated. Missing data rates did not differ statistically between severe mental health difficulty groups, and pairwise deletion handled missing data.

Group comparisons utilised independent samples *t*-tests for normally distributed data and Mann–Whitney *U* tests for non-normally distributed or unevenly distributed data. Chi-square tests assessed associations between categorical variables. Hierarchical multiple regression was used to assess the extent to which the presence of a severe mental health difficulty diagnosis predicts lifespan. Kaplan Meier curves were used to measure the period of time elapsed between the first nephrology appointment and kidney failure or death, whichever event occurred first. The log rank test was used to compare the survival distributions between individuals with and without severe mental health difficulties, concerning the time elapsed between the first nephrology appointment and kidney failure/death. Cox regression analyses were used to assess the association between the severe mental health difficulty group and mortality/kidney failure. Estimated glomerular filtration rate (eGFR) at first clinic appointment was excluded as a variable from regressions due to high rates of missing data (52%). Models including eGFR are included in supplementary materials. Cohen’s *d* measured effect size, the significance level was set at *α* = 0.05.

## Results

### Rates of severe mental health difficulty diagnoses

Of the 5105 individuals in the cohort, 112 (2.2%) had a recorded severe mental health difficulty diagnosis, 95% CI 1.8–2.6%]. The most common diagnoses were bipolar disorder (*n* = 48), schizophrenia (*n* = 32) and psychotic disorder (*n* = 15). See Supplementary Table V for the full list of severe mental health difficulty diagnoses.

### Rates of psychotropic medication prescriptions

In the cohort, 160 individuals were prescribed antipsychotics, with 33 of them receiving lithium. Fourteen individuals were prescribed antipsychotics for dementia/delirium. Additionally, 75 were prescribed mood stabilisers/anticonvulsants, of whom 35 received anticonvulsants for epilepsy. Out of the 912 people prescribed antidepressants, 63 had severe mental health difficulty diagnoses. Assessing why individuals were prescribed antidepressants was beyond the scope of this study. Six individuals were prescribed depot injections.

### Demographic differences

Individuals with severe mental health difficulties were less likely to be currently married (21.4%) compared to those without severe mental health difficulties (31.2%), *X*^*2*^(1, *N* = 2700) = 3.88, *p* = 0.049 (see Table 1). There was a significantly higher proportion of women in the severe mental health difficulty group compared to those without severe mental health difficulties, *X*^*2*^(1, *N* = 5102) = 6.95, *p* = 0.008. There was no significant difference in Deprivation Rank between those with and without severe mental health difficulties, *U* = 260,803.5 *z* = − 1.14, *p* = 0.255. There was no significant association between severe mental health difficulty group and ethnicity, *X*^*2*^(3, *N* = 4115) = 5.6, *p* = 0.13.

**Table 1:** Demographic and clinical characteristics of the cohort

	Total *N* = 5105	No SMHD *n* = 4993	SMHD *n* = 112
**Age at time of data extraction^**			
M (SD)	62.2 (17.1)	62.3 (17.2)	62.3 (17.2)
Range	21–99	21–99	25–86
**Outcome, n (%)**			
Alive	3842 (75.1%)	3754 (75.2%)	80 (71.0%)
Deceased	1273 (24.9%)	1239 (24.8%)	32 (28.6%)
Lifespan in years *			
Mean (SD)	75.6 (12.9)	75.9 (12.8)	62.8 (10.6)
Range	26–99	26–99	41–84
Sex, n (%) *			
Male	2774 (54.2%)	2720 (54.5%)	47 (42.0%)
Female	2338 (45.7%)	2270 (45.5%)	65 (58.0%)
Not specified	3 (0.1%)	3 (0.1%)	0
Marital Status, n (%) *			
Married	1581 (30.9%)	1556 (31.2%)	24 (21.4%)
Divorced/separated	197 (3.8%)	192 (3.8%)	5 (4.5%)
Widowed	191 (3.7%)	190 (3.8%)	1 (0.9%)
Single	774 (15.1%)	749 (15%)	23 (20.5%)
Not specified	2257 (44.1%)	2306 (46.2%)	59 (52.7%)
**Ethnicity, n (%)**			
Asian	324 (6.3%)	318 (6.4%)	6 (5.4%)
Black	1266 (24.8%)	1228 (24.6%)	37 (33%)
White	2334 (45.6%)	2278 (45.6%)	50 (44.6%)
Other	198 (3.9%)	197 (3.9%)	1 (0.9%)
Not specified	993 (19.4%)	927 (19.5%)	18 (16.1%)
**Index of Multiple Deprivation Decile**			
Mean (SD)	5.13 (2.52)	5.13 (2.55)	4.86 (2.54)
Range	1–10	1–10	1–10
Missing data, *n* (%)	24 (4.7%)	24 (4.7%)	0
**Received RRT, n (%)**	1667 (32%)	1639 (32.8%)	43 (38.4%)
Of those ever received Transplant*	740 (44.4%)	731 (44.6%)	9 (20.9%)
Of those ever received Haemodialysis	1378 (82%)	1339 (81.7%)	39 (90.7%)
Of those ever received Peritoneal Dialysis*	535 (32.1%)	528 (32.2%)	7 (16.2%)
**On treatment for concurrent conditions, n (%)**			
Hypertension	4619 (90.3%)	4511 (90.3%)	99 (88.4%)
Diabetes	1703 (33.3%)	1168 (33.4%)	34 (30.4%)
**BMI at time of first clinic appointment**			
Mean (SD)	28.48 (5.4)	28.47 (5.4)	29.07 (5.9)
Range	14.42–41.9	14.42–41.9	14.9–41.6
Missing data, *n* (%)	1164 (22.8%)	1133 (22.19%)	26 (23.21%)

Abbreviations: BMI = body mass index; RRT = renal replacement therapy; SD = standard deviation; SMHD = severe mental health difficulty

^Ages of those who were not deceased at time of data extraction

*Statistically significant difference between those with and without SMHDs

### Differences in lifespan

Individuals with severe mental health difficulty diagnoses had a significantly shorter lifespan (*M* = 62.8 years, *SD* = 10.59) compared to those without a severe mental health difficulty diagnosis (*M* = 75.9 years, *SD* = 12.77), *t*(1269) = 5.752, *p* < 0.001, Cohen’s *d* = 1.03. The mean difference in lifespan between those with and without a severe mental health difficulty diagnosis was 13.1 years, 95% CI 8.62, 17.55]. Rates of recorded causes of death (categorised into cardiovascular, infection, treatment withdrawal or other) did not differ between those with and without a severe mental health difficulty, *X*^*2*^(4, *N* = 1271) = 1.5.09,* p* = 0.278 (see Supplementary Table VI for breakdown of causes of death).

Step 1 of the hierarchical regression including demographic and clinical variables (see Table 2), explained 20.3% variance in lifespan *F*(10,649) = 16.57, *p* < 0.001. At Step 2, adding severe mental health difficulty contributed an additional 2.2% of variance in lifespan, *F*(11,648) = 17.09, *p* < 0.001. After controlling for demographic and clinical variables, having a severe mental health difficulty diagnosis was associated with a decrease in lifespan of 12.92 years.

**Table 2:** Hierarchical regression analysis of lifespan

	*B*	*(SE)*	Beta	*t*	*p*
**Step 1**					
Sex (1 = female, 0 = male)	0.61	(0.92)	0.03	0.664	0.507
Deprivation	0.65	(0.19)	0.13	3.414	0.001*
Asian ethnicity (1 = yes, 0 = no)	− 0.51	(2.17)	− 0.01	− 0.195	0.845
Black ethnicity (1 = yes, 0 = no)	0.73	(2.23)	0.03	0.327	0.744
White ethnicity (1 = yes, 0 = no)	− 2.4	(2.16)	− 0.09	− 1.109	0.268
Marital status (1 = currently married, 0 = not currently married)	0.43	(0.95)	0.02	0.454	0.650
Received RRT (1 = yes, 0 = no)	− 9.16	(0.99)	− 0.33	− 9.180	< 0.001*
BMI	− 0.21	(0.09)	− 0.9	− 2.370	0.018*
Diabetes (1 = yes, 0 = no)	− 3.21	(1.0)	− 0.12	− 3.195	0.001*
Hypertension (1 = yes, 0 = no)	7.25	(1.57)	0.17	4.569	< 0.001*
**Step 2**					
Sex (1 = female, 0 = male)	0.49	(0.91)	0.02	0.53	0.59
Deprivation	0.65	(0.19)	0.13	3.45	0.001*
Asian ethnicity (1 = yes, 0 = no)	− 0.685	(2.59)	− 0.014	− 0.265	0.791
Black ethnicity (1 = yes, 0 = no)	0.47	(2.21)	0.02	0.21	0.833
White ethnicity (1 = yes, 0 = no)	− 2.6	(2.14)	− 0.1	− 1.2	0.224
Marital status (1 = currently married, 0 = not currently married)	0.327	(0.94)	0.013	0.349	0.728
Received RRT (1 = yes, 0 = no)	− 9.07	(0.99)	− 0.33	− 9.2	< 0.001*
BMI	− 0.2	(0.09)	− 0.084	− 2.32	0.021*
Diabetes (1 = yes, 0 = no)	− 3.26	(0.993)	− 0.119	− 3.28	0.001*
Hypertension (1 = yes, 0 = no)	7.18	(1.57)	0.165	4.58	< 0.001*
SMHD diagnosis (1 = yes, 0 = no)	− 12.92	(3.04)	− 0.147	− 4.24	< 0.001*

Abbreviations: BMI = body mass index; RRT = renal replacement therapy; SMHD = severe mental health difficulty

*Statistically significant difference between those with and without SMHDs

### Time from first clinic appointment to death or kidney failure

**Fig. 2:**
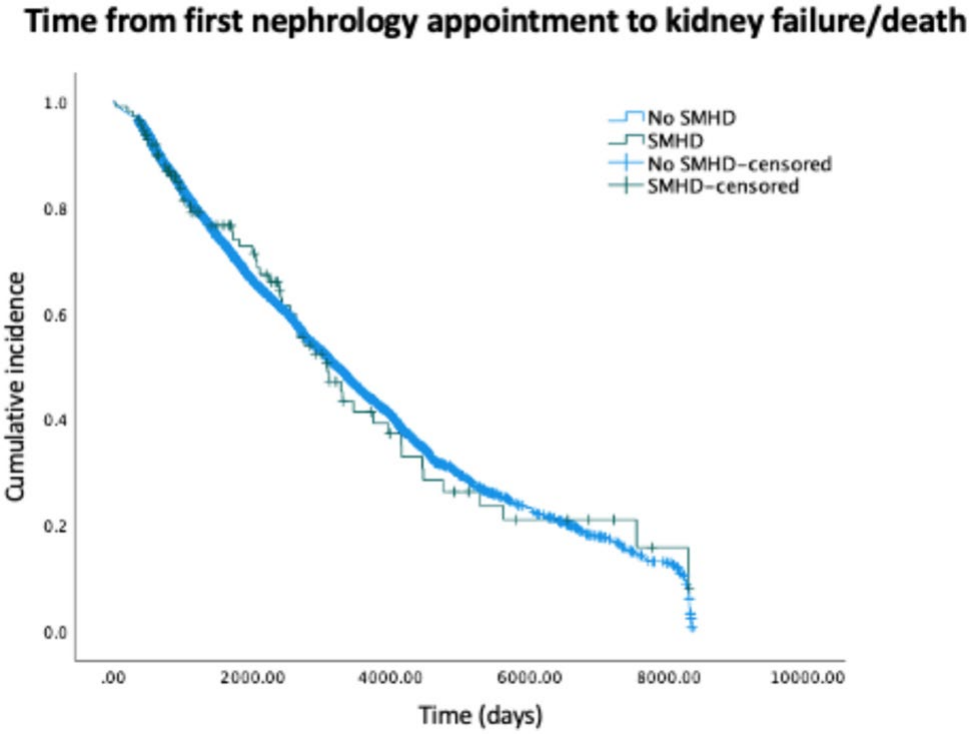
Kaplan Meier Curve illustrating time from first nephrology appointment to kidney failure/death

No statistically significant difference was found between length of time to death/kidney failure for those with (*Md* = 1766 days, *IQR* = 795.25–3101.0) and without severe mental health difficulties (*Md* = 1680 days, *IQR* = 865.0–3070.0), *χ*^2^(1) = 0.001, *p* = 0.980). See Fig. 2 for Kaplan Meier Curve.

As illustrated in Table 3, Crude and adjusted Cox regression analyses (*n* = 1257) indicated no association between the severe mental health difficulty group and all-cause mortality/kidney failure, before or after adjusting for potential confounders.

**Table 3:** Multiple Cox regression models of all-cause mortality/kidney failure

	Crude model		Adjusted model	
	Hazard ratio (95% CI)	*p*	Hazard ratio (95% CI)	*p*
SMHD diagnosis (1 = yes, 0 = no)	0.82 (0.58–1.2)	0.265	1.267 (0.58–1.6)	0.228
Age at first appointment			1.03 (1.017–1.035)	< 0.001*
Sex			1.069 (0.7–1.2)	0.270
Deprivation			0.967 (0.94–1.02)	0.007*
White ethnicity (1 = yes, 0 = no)			0.919 (0.4–9.05)	0.553
Black ethnicity (1 = yes, 0 = no)			0.880 (0.51–1.1)	0.386
Asian ethnicity (1 = yes, 0 = no)			0.815 (0.6-0.97)	0.214
Marital status (1 = currently married, 0 = not currently married)			0.899 (0.46–1.5)	0.084
Received RRT (1 = yes, 0 = no)			5.820 (4.5–8.9)	< 0.001*
BMI			0.987 (9.5–1.1)	0.029*
Diabetes (1 = yes, 0 = no)			1.155 (0.94–1.23)	0.024*
Hypertension (1 = yes, 0 = no)			1.045 (1.02–1.07)	0.845

Abbreviations: BMI = body mass index; RRT = renal replacement therapy; SMHD = severe mental health difficulty

*Statistically significant difference between those with and without SMHDs

### Differences in clinical health data

People with severe mental health difficulties had significantly lower eGFR at the time of their first nephrology appointment, and had their first appointment at a younger age (see Table 4). There were no statistically significant differences between groups with regard to other clinical data.

**Table 4:** Clinical health data of those with and without SMHDs

Variable	No SMHD	SMHD	Statistic	*p* value
eGFR (ml/min/1.73m^2^) at first clinic appointment, *Md (IQR)*	38 (27.5–53)* *n* = 2404	29 (23.5–42.5)* *n* = 57	*U* = 55,053.5 *z* = − 2.54	0.011*
HbA1c (mmol/mol), *Md (IQR)*	52 (37–72) *n* = 229	42 (37–68) *n* = 24	*U* = 2032.0 *z* = − 1.8	0.07
Serum Total Cholesterol (mmol/mol), *M (SD)*	4.92 (1.5) *n* = 476	4.8 (1.2) *n* = 37	*t*(511) = -0.48 Cohen’s *d* = -0.083	0.63
Haemoglobin (g/dL),* M (SD)*	11.43 (2.36) *n* = 465	11.95 (2.05) *n* = 59	*t*(522) = 1.62 Cohen's *d* = 0.22	0.12
Albumin (g/L),* M (SD)*	39.7 (6.06) *n* = 2722	40.9 (5.9) *n* = 61	*t*(2781) = 1.47 Cohen's *d* = 0.19	0.14
Phosphate (mg/dL),* M (SD)*	1.21 (0.34) *n* = 2591	1.22 (0.38) *n* = 59	*t*(2648) = 0.4 Cohen's *d* = 0.05	0.69
Urine Albumin-Creatinine ratio (mg/g), *Md (IQR)*	25 (0.93–53.3) *n* = 376	43 (1.0–50.0) *n* = 9	*U* = 1598.0, *z* = − 0.17	0.87
Protein-Creatinine ratio (mg/mg), *Md (IQR)*	110 (50.3–381.4) *n* = 1413	99 (24.23–261.1) *n* = 34	*U* = 21,526.5 *z* = − 1.04	0.3
Hypertension, *n* (%)	4511 (90.3%)	99 (88.4%)	*X*^*2*^(1, N = 5105) = 0.458	0.5
Diabetes, *n* (%)	1168 (33.4%)	34 (30.4%)	*X*^*2*^(1, N = 5105) = 0.477	0.49
BMI, *M (SD)*	28.47 (5.4) *n* = 3,972	29.07 (5.9) *n* = 86	*t*(3944) = 1.02 Cohen's *d* = 0.114	0.301
Age (years) at kidney failure, *M (SD)*	54.02 (16.3) *n* = 1624	52.49 (12.18) *n* = 43	*t*(1665) = 0.611 Cohen's *d* = − 0.202	0.54
Age (years) at time of first clinic appointment, *M (SD)*	56.13 (18.27)* *n* = 4974	51.01 (12.55)* *n* = 112	*t*(5084) = − 2.95 Cohen's *d* = − 0.282	0.003*
Time (months) elapsed between first appointment and initiating RRT, *Md (IQR)*	49 (27–97) *n* = 1061	50 (17.3–95) *n* = 35	*U* = 24,000.0, *z* = − 0.286	0.775

Abbreviations: CKD = chronic kidney disease; eGFR = estimated glomerular filtration rate; g/dL = grams per decilitre, mg/dL = milligrams per decilitre; mmol/mol = millimoles per mole; HBA1C = Glycated haemoglobin; IQR = inter quartile range; M = mean; Md = Median; SD = standard deviation; SMHD = severe mental health difficulty

*Statistically significant difference between those with and without SMHDs

### Hospital inpatient admissions

There were no statistically significant differences in those with and without severe mental health difficulties concerning rates of inpatient admissions to the hospital, *U* = 261,044.5, *z* = − 1.36, *p* = 0.175; or the number of days spent as an inpatient, *U* = 264,525.5, *z* = − 1.12, *p* = 0.263 (see Table 5). People with severe mental health difficulties had higher rates of admissions to the ICU compared to those without severe mental health difficulties,* U* = 259,487.5, *z* = − 2.87, *p* = 0.004, and spent more days in ICU, *U* = 267,631.0, *z* = − 2.646, *p* = 0.008. People with severe mental health difficulties also had higher rates of emergency admissions, *U* = 261,747.5, *z* = − 2.08, *p* = 0.038.

**Table 5:** Inpatient admissions to King’s College Hospital for those with and without SMHDs

Variable	No SMHD	SMHD
**Number of inpatient hospital admissions, *Md (IQR)***	**0 (3)**	**0 (3)**
0 admissions	59.7%	55.4%
1–2 admissions	15.2%	16.1%
3–4 admissions	9.1%	8%
5 + admissions	16%	20.5%
**Total days spent as an inpatient, *Md (IQR)***	**0 (8)**	**0 (15)**
0 days	61.8%	59.8%%
1–5 days	9.1%	5.4%
6–20 days	12.1%	11.6%
20–40 days	6.5%	5.4%
40 + days	10.5%	17.9%
**Number of ICU admissions, *Md (IQR)***	0 (0)*	0 (0)*
0 admissions	92.7%	85.7%
1–2 admissions	5.7%	8.9%
3–4 admissions	1.1%	3.6%
5 + admissions	0.5%	1.8%
**Total days spent as inpatient in ICU, *Md (IQR)***	**0 (0)**	**0 (0)**
0 days	97.1%	92.9%
1–20 days	1.4%	2.7%
21–50 days	0.7%	2.7%
40 + days	0.7%	1.8%
**Number of emergency hospital admissions, *Md (IQR)***	0 (0)*	0 (0)*
0 admissions	90.5%	86%
1 admission	7.1%	11.2%
2 admissions	2.4%	2.8%

Abbreviations: IQR = inter quartile range; Md = Median; SD = standard deviation; SMHD = severe mental health difficulty

*Statistically significant difference between those with and without SMHDs

### Renal replacement therapy modalities

There was no statistically significant difference in rates of RRT between those with and without severe mental health difficulties, *X*^*2*^(1, *N* = 5105) = 1.71,* p* = 0.19. Among people who received RRT, those with severe mental health difficulties were less likely to have ever received a kidney transplant (20.9%) compared to those without a severe mental health difficulty (45.0%), *X*^*2*^(1, *N* = 1667) = 9.79, *p* = 0.002. People with severe mental health difficulties were also less likely to have ever received peritoneal dialysis (16.3%) compared to those without severe mental health difficulties (32.4%), *X*^*2*^(1, N = 1667) = 5.0, *p* = 0.025.

### Clinic appointment attendance

Among individuals not requiring RRT, those with severe mental health difficulties had higher rates of appointment non-attendance (*Md* = 18.3%, *IQR* = 35%) compared to those without severe mental health difficulties (*Md* = 11.1%, *IQR* = 30%), *U* = 23,464.0, *z* = − 2.06, *p* = 0.039, *r* = 0.05. However the effect size was small. For patients requiring RRT, there were no significant differences between those with (*Md* = 11.5%, *IQR* = 40%) and without severe mental health difficulties (*Md* = 10.8%, *IQR* = 18%) with regard to rates of attendance, *U* = 18,965.0, *z* = − 0.46, *p* = 0.647, *r* = 0.01.

### Adherence on haemodialysis

Among patients on haemodialysis (*n* = 845), those with severe mental health difficulties had a higher proportion of shortened sessions, greater mean weight loss during haemodialysis sessions, and a higher proportion of serum potassium and phosphate levels outside normal ranges (see Table 6). There were no other statistically significant differences in haemodialysis data between those with and without severe mental health difficulties (see Table 6).

**Table 6:** Haemodialysis data of individuals with and without SMHDs

Variable	No SMHD *n* = 818	SMHD *N* = 27	Statistic	*p* value
Months on Dialysis, *Md (IQR)*	31.2 (4.9–59.6)	34.8 (6.1–58.7)	*t*(894) = − 0.37 Cohen's *d* = 0.06	0.71
Proportion missed HD sessions*, Md (IQR)*	0.01% (0.02)	0.01% (0.06)	*U* = 9806 *z* = − 1.5	0.134
Proportion minutes HD shortfall*, Md (IQR)*	1.04% (3.09)	2.29% (5.45)	*U* = 8938 *z* = − 2.12	0.034*
Proportion of potassium values ≥ 6.0 mmol/l*, Md (IQR)*	7.14% (20.47)	16.67% (33.39)	*U* = 9082.0 *z* = − 2.0	0.045*
Proportion of phosphate values ≥ 2.0 mmol/l*, Md (IQR)*	5.68% (19.2)	15.3% (40.61)	*U* = 8278.0 *z* = − 2.6	0.009*
Mean weight loss as a percentage of body weight*, Md (IQR)*	3.17% (3.33)	5.09% (8.08)	*U* = 7969.5 *z* = − 2.84	0.005*
Mean pre-dialysis systolic BP (mmHg), *M (SD)*	143.8 (18.3)	143.26 (15.67)	*t*(894) = − 0.151 Cohen's *d* = − 0.03	0.88
Mean pre-dialysis diastolic BP, *M (SD)*	74.4 (11.68)	76.47 (9.9)	*t*(894) = 0.912 Cohen's *d* = 0.17	0.362

Abbreviations: CKD = chronic kidney disease; eGFR = estimated glomerular filtration rate; g/dL = grams per decilitre, mg/dL = milligrams per decilitre; mmol/mol = millimoles per mole; HBA1C = Glycated haemoglobin; IQR = inter quartile range; M = mean; Md = Median; SD = standard deviation; SMHD = severe mental health difficulty.

*Statistically significant difference between those with and without SMHDs

## Discussion

Our findings demonstrate that, among people with CKD, those with severe mental health difficulties have shorter lifespans, and also may receive suboptimal kidney care. In this cohort of 5105 people with CKD, 2.2% had a recorded severe mental health difficulty diagnosis. This is higher than the 1% observed in primary care in England [18], likely due to the higher risk for developing CKD in people with severe mental health difficulties [3]. The average lifespan of people with a severe mental health difficulty diagnosis was over a decade shorter than those without a severe mental health difficulty. There was no difference in the number of inpatient admissions; however those with severe mental health difficulties had higher rates of emergency and ICU admissions. People with severe mental health difficulties had more advanced CKD at the time of their first nephrology appointment, but there were no statistically significant differences between groups concerning other laboratory test results, BMI, or rates of diabetes and hypertension. There were no statistically significant differences between groups in the rates of kidney failure, age at onset of kidney failure, or the amount of time elapsed between their first appointment and death/kidney failure. However, for patients on RRT, those with severe mental health difficulties were less than half as likely to have ever received a kidney transplant, or to have received peritoneal dialysis. Among subjects not requiring RRT, those with severe mental health difficulties had lower rates of appointment attendance. However, regarding patients established on RRT there was no difference in attendance rates between those with and without severe mental health difficulties. Patients with severe mental health difficulties receiving haemodialysis had a higher proportion of shortened dialysis sessions, and findings indicate they had poorer adherence to their medication, fluid and dietary treatment requirements.

Population studies show that people with severe mental health difficulties die 10–20 years younger than those without severe mental health difficulties, and that higher rates of physical illness and deprivation are leading causes of this disparity [19, 20]. However, in this study people with severe mental health difficulties had comparable rates of physical illness and deprivation, possibly because they are also risk factors for CKD, and severe mental health difficulty was associated with reduced lifespan even when controlling for demographic and clinical variables. Possible contributors to premature mortality in people with CKD and concurrent severe mental health difficulties include inequitable access to quality healthcare, side effects of psychotropic medications, treatment adherence, limited social support, smoking, substance use, and suicide [21]. Although causes of death did not differ statistically between those with and without severe mental health difficulties, 60% of these data were missing, and there were no recorded deaths relating to suicide or accidents. As there are high rates of suicide attempts in people with CKD [22], further research regarding rates of deaths by suicide in people with CKD and severe mental health difficulties is needed.

People with severe mental health difficulties had their first nephrology appointment at a later stage of CKD, despite being younger at the time of this appointment. Consistent with previous research, those with severe mental health difficulties had higher rates of emergency and ICU admissions [23, 24]. Some people with severe mental health difficulties may have additional difficulty engaging in healthcare due to anxiety, depression, low motivation and communication difficulties [9]. Stigma can also lead to delayed medical care for people with severe mental health difficulties, as clinicians are more likely to minimise and dismiss their concerns [9, 25]. Emergency and ICU admissions for people with severe mental health difficulties may be reduced by conducting physical health clinics within psychiatric services, increasing the adoption of care plans, and tailoring interventions to manage physical health issues in people with severe mental health difficulties [1, 9]. Results indicate that people with severe mental health difficulties on haemodialysis had poorer adherence to medication, diet and fluid treatment requirements. This supports evidence that some people with CKD and severe mental health difficulties require additional support for adherence, tailored to the needs of each individual [9].

People with severe mental health difficulties were less likely to receive a kidney transplant, consistent with previous research [1, 7, 10]. This may be due to clinician concerns regarding adherence, drug interactions and limited social support, as well as clinician’s stigma towards people with severe mental health difficulties [9]. Research indicates that people with severe mental health difficulties have comparable outcomes post-transplant, but are less likely to receive an evaluation appointment for kidney transplant [3]. Thus, efforts should be made to ensure people with severe mental health difficulties are not being unfairly discriminated against, and that they are offered the optimal treatment in the context of their physical health, capacity, available support, and treatment preferences.

Although subjects with severe mental health difficulties had lower levels of kidney function at the time of their first nephrology appointment, they had comparable rates of kidney failure, and comparable time between their first appointment and death/kidney failure. This may be due to effective management of lithium medication with input from nephrology, and the high level of support provided to people with severe mental health difficulties by the centre’s psychosocial multidisciplinary teams, which include liaison psychiatry, psychology, social work, and a mental health nurse. As most renal services lack these supports [26], outcomes for people with severe mental health difficulties may be poorer in other settings.

Qualitative research indicates that kidney care should be tailored to meet the specific needs of each person with a severe mental health difficulty, in order to support adherence, reduce treatment burden and optimise the management of their concurrent physical and mental health conditions [9, 11]. Given the complex interplay of risk and protective factors for this population, improving outcomes will require a comprehensive system of coordinated support provided at each stage of care. Safe and effective treatment for people with CKD and severe mental health difficulties also requires integrated physical and mental health care, whereby renal teams are adequately staffed with mental health professionals [9, 11].

This study includes a large sample of a diverse group of individuals with CKD, but data were limited to a single renal unit population residing in urban/suburban settings. The cohort only included those engaged with renal services, excluding individuals with undiagnosed CKD who account for 30% of CKD cases in the United Kingdom (UK) [27]. Furthermore, people with severe mental health difficulties are less likely to be engaged in kidney care [28]. Data from primary care may capture those with CKD who are not receiving specialised treatment, and thus may have poorer outcomes. Due to varying accuracy in EHCRs, some severe mental health difficulty diagnoses may have been misidentified. Findings may also have been influenced by unobserved confounders caused by systematic biases in measurement, such as the coding behaviour of clinicians, the person’s health status, or the level of contact they had with services. Due to the relatively small number of people with severe mental health difficulties identified, the study was not sufficiently powered to stratify results based on severe mental health difficulty subtype, or to detect small differences between groups. Research with a larger population of people with severe mental health difficulties may provide more nuanced insights. As the study period ended in 2019 before the COVID-19 pandemic, further research is needed to assess the impact of recent changes in care practices on outcomes for those with severe mental health difficulties.

Future research should prioritise identifying risk and protective factors for people with CKD and concurrent severe mental health difficulties, to identify those most at risk and inform targeted interventions. Potential predictors which were not included in the current study include smoking, diet, physical activity, suicide, substance use, and the dose and duration of psychotropic medication. The impact of healthcare provider stigma on quality of kidney care for people with severe mental health difficulties should be investigated, particularly regarding access to transplant. Research is also needed to determine the impact of specific social, community and hospital supports on outcomes for people with CKD and concurrent severe mental health difficulties.

## Conclusion

In a cohort of 5105 of people with CKD, 2.2% had a recorded severe mental health difficulty diagnosis. The average lifespan of individuals with severe mental health difficulties was over a decade shorter than those without a severe mental health difficulty diagnosis. In a centre which provides a high level of support to people with severe mental health difficulties, those with severe mental health difficulties had comparable rates of kidney failure to those without severe mental health difficulties. However, findings indicate that people with severe mental health difficulties may receive suboptimal kidney healthcare compared to those without severe mental health difficulties. For example, people with severe mental health difficulties had more advanced CKD at the time of their first nephrology appointment, and were less likely to receive a kidney transplant. People with severe mental health difficulties also had higher rates of emergency and ICU admissions. Results indicate that people with severe mental health difficulties require additional support to follow treatment requirements while on RRT. Findings underscore the need for tailored interventions which prevent premature mortality and improve care for people with CKD and severe mental health difficulties.

## Supplementary Information

Below is the link to the electronic supplementary material.

Supplementary file1 (DOCX 35 KB)

## Data Availability

The data underlying this article cannot be shared publicly for confidentiality reasons.
