# Supplementary material for: Premature mortality and disparities in kidney healthcare for people with chronic kidney disease and severe mental health difficulties
Source: J Nephrol. 2024 Nov 2;37(9):2609–20. doi: 10.1007/s40620-024-02103-6 (PMC11663822; doi:10.1007/s40620-024-02103-6)
Supplement: Supplementary file 1 — Supplementary file1 (DOCX 35 KB) [file 40620_2024_2103_MOESM1_ESM.docx]

# Supplementary materials

| Supplementary Table I. Valid ranges of clinically probably values | | |
| --- | --- | --- |
| **Variable** | **Valid range** | **n of clinically improbable values** |
| HbA1c (mmol/mol) | 20-100 | 0 |
| Serum Total Cholesterol (mmol/mol) | 1.5-15 | 0 |
| Haemoglobin (g/L) | 30-200 | 0 |
| Albumin (g/L) | 5-60 | 0 |
| Phosphate (mg/dL) | 0.1-4.0 | 0 |
| Urine Albumin-Creatinine ratio (mg/mmol) | 0-15,000 | 0 |
| Protein-Creatinine ratio (mg/mmol) | 0-15,000 | 0 |
| Height (m) | 1-3 | 4 |
| Weight (kg) | 30 - 160 | 27 |
| BMI (kg/m^2^) | 12 - 42 | 31 |
| Lifespan (years) | 0 - 110 | 2 |
| Abbreviations: *CKD = chronic kidney disease; eGFR = estimated glomerular filtration rate; g/dL = grams per decilitre, mg/dL = milligrams per decilitre; mmol/mol = millimoles per mole; HBA1C = Glycated haemoglobin; IQR = inter quartile range; M = mean; Md = Median; SD = standard deviation; SMHD = severe mental health difficulty* | | |

| Supplementary Table II. Extraction of demographic characteristics and outcome variables | | |
| --- | --- | --- |
| Variable | **Description** | **Source** |
| Sex | Coded as binary “male” or “female” in database. Other gender classifications were not available. | Renalware |
| Ethnicity | Self-identified ethnicity, categorized as White, Black, Asian, or other (including "Mixed"). | Renalware |
| Age (years) at time of data extraction | Calculated using DOB | Renalware |
| Deprivation | Calculated by matching postcodes to the UK Index of Multiple Deprivation (IMD) 2019 area scores ^21^. IMD quantifies relative deprivation within distinct geographic areas in England, categorizing them into deciles ranging from 1 (most deprived) to 10 (least deprived). This measure encompasses income, employment, education, health, crime, barriers to housing and services, and the living environment. | Renalware |
| Marital status | Categorised into “currently married” or “not currently married”. | Renalware |
| Outcome | Categorised as “deceased” or “alive” based on presence/absence of recorded date of death. | Renalware |
| Lifespan (years) | Calculated using DOB and date of death. | Renalware |
| Age of onset of kidney failure | Calculated using DOB and date of kidney failure | Renalware |
| Body Mass Index (BMI) | Calculated using each person’s first recorded weight/height^2^ (kg/m^2^). | Renalware |
| Date first seen by Nephrology | Date of first clinic appointment with Nephrology. | Renalware |
| Cause of death | Categorized into four groups: "cardiovascular", "infection", "treatment withdrawal", and "other". | Renalware |
| Time from first appointment to kidney failure or death | Calculated using person’s date of kidney failure or death (earliest event), and subtracting the date of their first nephrology appointment | Renalware |
| Medication prescriptions | Based on if person ever had a recorded prescription of antipsychotics, mood stabilisers, and antidepressants. | Renalware |
| Presence of diabetes/hypertension | Based on if person had ever had a recorded prescription of diabetic/hypertensive medications. | Renalware |
| RRT received | Whether individuals had ever received a kidney transplant, peritoneal dialysis and haemodialysis. | Renalware |
| Number of hospital admissions | Total number of inpatient and ICU discharge summaries. Total number of admissions through the emergency department were also calculated. | KCH EHCR |
| Days spent as an inpatient | Calculated based on intake and discharge dates. | KCH EHCR |
| Laboratory test results | Taken closest to and within three months of their first clinic visit with Nephrology. Included measures of HbA1c (mmol/mol), Serum Total Cholesterol (mmol/mol), Haemoglobin (g/dL), Albumin (g/L), Phosphate (mg/dL), Urine Albumin-Creatinine ratio (mg/g), Urine Protein-Creatinine ratio (mg/mg). | KCH EHCR |
| eGFR | eGFR (mls/min/1.73m^2^) calculated from serum creatinine measure, taken closest to and within three months of their first clinic visit with Nephrology. Calculation based on CKDFR 2009 guidelines with no ethnicity adjustment^22^ | KCH EHCR |
| Appointment non-attendance | Calculated by dividing the number of nephrology clinic appointments attended by the number of appointments offered | KCH EHCR |
| Months on haemodialysis | Total number of months each person spent on haemodialysis | Renalware |
| Proportion missed haemodialysis sessions | The number of missed haemodialysis sessions, divided by the total number of sessions offered | Renalware |
| Proportion haemodialysis shortfall | The average percentage of missed minutes of haemodialysis sessions for each person | Renalware |
| Serum potassium values ≥ 6.0 mmol/l | The percentage of serum potassium values ≥ 6.0 mmol/l, for those on haemodialysis. Values outside this range indicate that the person may not fully adhere to dietary or treatment requirements. | Renalware |
| Serum phosphate values ≥ 2.0 mmol/l | The percentage of serum phosphate values ≥ 2.0 mmol/l, for those on haemodialysis. Values outside this range indicate that the person did not fully adhere to medication (phosphate binders) or dietary or treatment requirements. | Renalware |
| Mean weight loss as a percentage of body weight | Mean weight loss during haemodialysis session, divided by each person’s body weight. A higher value indicates that the person consumed larger amounts of fluid between sessions. | Renalware |
| Mean pre-dialysis systolic BP | Mean systolic blood pressure before haemodialysis | Renalware |
| Mean pre-dialysis diastolic BP | Mean diastolic blood pressure before haemodialysis | Renalware |
| *Abbreviations: BP=Blood Pressure; DOB=date of birth; ECHRs=electronic healthcare records; eGFR=Estimated Glomerular Filtration Rate; KCH=King’s College Hospital; g/dL=grams per decilitre, mg/dL=milligrams per decilitre; mmol/mol=millimoles per mole; RRT=renal replacement therapy* | | |

List of searched terms using Cogstack

**ICD F20-F29**

Schizo*

Delusional

Psycho*

Bipol*

BP

BPAD

Mani*

Hypomania

Depress*

|  |  |  |  |
| --- | --- | --- | --- |

**ICD G40-G43**

Epil*

Seizure*

atonic

clonic

myoclonic

tonic*

**ICD F00- F02.8**

Dementia

Alzheimer*

Parkinson*

Huntington*

Pick

Creutzfeldt-Jakob

## Hierarchical regression analysis of lifespan including eGFR

Step 1 of the hierarchical regression including demographic and clinical variables (see Supplementary 7), explained 31.6% variance in lifespan *F*(11,570)=23.98, *p*<.001). Step 2, adding SMHD, contributed an additional 2.8% of variance in lifespan *F*(11,569)=24.92, *p*<.001. After controlling for demographic and clinical variables, having a SMHD diagnosis was associated with a decrease in lifespan of 13.54 years.

| **Supplementary Table III**. Hierarchical regression analysis of lifespan including eGFR | | | | | |
| --- | --- | --- | --- | --- | --- |
|  | ***B*** | ***(SE)*** | ***Beta*** | ***t*** | ***p*** |
| **Step 1** |  |  |  |  |  |
| Sex (1 = female, 0 = male) | .186 | (.91) | .007 | .204 | .839 |
| Deprivation | .593 | (.18) | .118 | 3.136 | .002 |
| White ethnicity (1 = yes, 0 = no) | .717 | (2.16) | .028 | .331 | .74 |
| Black ethnicity (1 = yes, 0 = no) | 3.429 | (2.22) | .123 | 1.540 | .124 |
| Asian ethnicity (1 = yes, 0 = no) | 1.530 | (2.59) | .032 | .589 | .556 |
| Marital status (1 = yes, 0 = no) | -.124 | (.94) | -.005 | -.131 | .895 |
| Received RRT (1 = yes, 0 = no) | -13.33 | (1.07) | -.487 | -12.397 | <.001 |
| BMI | -.182 | (.08) | -.076 | -2.090 | .037 |
| Diabetes (1 = yes, 0 = no) | -4.129 | (.99) | -.151 | -4.133 | <.001 |
| Hypertension (1 = yes, 0 = no) | 4.129 | (1.60) | .095 | 2.578 | .01 |
| eGFR at first appointment | -.199 | (.02) | -.389 | -9.707 | <.001 |
| **Step 2** |  |  |  |  |  |
| Sex (1 = female, 0 = male) | .029 | (.89) | .001 | .032 | .974 |
| Deprivation | .588 | (.18) | .117 | 3.175 | .002 |
| White ethnicity (1 = yes, 0 = no) | .584 | (2.12) | .023 | .276 | .783 |
| Black ethnicity (1 = yes, 0 = no) | 3.207 | (2.18) | .115 | 1.470 | .142 |
| Asian ethnicity (1 = yes, 0 = no) | 1.393 | (2.54) | .029 | .548 | .584 |
| Marital status (1 = yes, 0 = no) | -.260 | (.92) | -.010 | -.282 | .778 |
| Received RRT (1 = yes, 0 = no) | -13.36 | (1.05) | -.488 | -12.673 | <.001 |
| BMI | -.173 | (.08) | -.072 | -2.026 | .043 |
| Diabetes (1 = yes, 0 = no) | -4.210 | (.97) | -.154 | -4.299 | <.001 |
| Hypertension (1 = yes, 0 = no) | 3.950 | (1.57) | .091 | 2.516 | .012 |
| eGFR at first appointment | -.205 | (.02) | -.401 | -10.189 | <.001 |
| SMHD diagnosis (1 = yes, 0 = no) | -14.78 | (2.99) | -.168 | -4.941 | <.001 |
| Abbreviations: BMI= body mass index; eGFR= estimated glomerular filtration rate; RRT = renal replacement therapy; SMHD = severe mental health difficulty | | | | | |

## Cox regression analyses including eGFR

As illustrated in Supplementary Table 2, Crude and adjusted Cox regression analyses (n=1011) indicated no association between SMHD group and all-cause mortality/kidney failure, before or after adjusting for potential confounders.

| **Supplementary Table IV**. Multiple Cox regression models of all-cause mortality/kidney failure | | | | |
| --- | --- | --- | --- | --- |
|  | **Crude model** | | **Adjusted model** | |
|  | **Hazard ratio (95% CI)** | ***p*** | **Hazard ratio (95% CI)** | ***p*** |
| SMHD diagnosis (1 = yes, 0 = no) | .82 | .265 | .958  (.58 – 1.57) | .865 |
| Age at first appointment |  |  | 1.023  (1.01 – 1.03) | <.001 |
| Sex |  |  | .96  (.81 – 1.15) | .707 |
| Deprivation |  |  | .981  (.94 – 1.02) | .321 |
| White ethnicity (1 = yes, 0 = no) |  |  | .727  (.5 – 1.1) | .094 |
| Black ethnicity (1 = yes, 0 = no) |  |  | .729  (.5 – 1.0) | .104 |
| Asian ethnicity (1 = yes, 0 = no) |  |  | .599  (.38 - .95) | .030 |
| Marital status (1 = yes, 0 = no) |  |  | 1.126  (.94 – 1.3) | .186 |
| Received RRT (1 = yes, 0 = no) |  |  | 6.94  (5.54 – 8.7) | <.001 |
| BMI |  |  | .974  (.96 - .99) | .002 |
| Diabetes (1 = yes, 0 = no) |  |  | 1.015  (.85 – 1.2) | .875 |
| Hypertension (1 = yes, 0 = no) |  |  | .829  (.46 – 1.5) | .536 |
| eGFR |  |  | .990  (.99 – 1.0) | <.001 |
| Abbreviations: BMI= body mass index; eGFR= estimated glomerular filtration rate; RRT = renal replacement therapy; SMHD = severe mental health difficulty | | | | |

| **Supplementary Table V** Rates of SMHD diagnoses and psychiatric medication prescriptions | | |
| --- | --- | --- |
| **SMHD diagnoses** | **n** | **% of cohort** |
| Bipolar disorder  Schizophrenia  Psychotic Disorder  Of those steroid induced psychosis  Depression with psychotic features  Schizoaffective disorder  Schizoaffective disorder and Bipolar Disorder  Schizophrenia and Bipolar Disorder | 48  31  15  2  7  5  3  1 | 0.94%  0.61%  0.29%  0.04%  0.14%  0.10%  0.06%  0.02% |
| **Total** | **112** | **2.2%** |
| **Psychiatric medication prescriptions** | **n** | **%** |
| Mood stabilisers  Of those Lithium | 75  33 | 1.4%  0.6% |
| Antipsychotics | 160 | 3.1% |
| Depot injections | 6 | 0.1% |
| Antidepressants  Of those tricyclics | 912  251 | 17.8%  4.5% |
| *Abbreviations: SMHD=severe mental health difficulty* | | |

| **Supplementary Table VI.** Causes of death of people with and without SMHDs | | |
| --- | --- | --- |
| **Cause of death** | **No SMHD**  **n (% of deceased)** | **SMHD**  **n (% of deceased)** |
| Cardiovascular | 164 (13.2%) | 6 (18.8%) |
| Infection | 144 (11.6%) | 6 (18.8%) |
| Treatment withdrawal | 78 (6.3%) | 3 (9.4%) |
| Other reasons | 106 (8.6%) | 4 (12.5%) |
| Unknown/missing | 747 (60.3%) | 13 (40.6%) |
| **Total** | **1239** | **32** |
| *Abbreviations: SMHD=severe mental health difficulty* | | |
